# Draft Genome Sequences of 43 Enterococcus faecalis and Enterococcus faecium Isolates from a Commercial Beef Processing Plant and Retail Ground Beef

**DOI:** 10.1128/MRA.00974-19

**Published:** 2019-10-17

**Authors:** Devin B. Holman, Katherine E. Gzyl, Rahat Zaheer, Tineke H. Jones, Tim A. McAllister

**Affiliations:** a Lacombe Research and Development Centre, Agriculture and Agri-Food Canada, Lacombe, Alberta, Canada; b Lethbridge Research and Development Centre, Agriculture and Agri-Food Canada, Lethbridge, Alberta, Canada; Loyola University Chicago

## Abstract

Here, we report the draft genome sequences of 36 Enterococcus faecalis and 7 Enterococcus faecium isolates recovered from a beef processing facility and retail ground beef. The beef processing facility samples were collected from beef carcasses, conveyor belts, and ground product.

## ANNOUNCEMENT

Enterococcus faecalis and Enterococcus faecium are commensal microorganisms present in the gastrointestinal tract of both humans and cattle; therefore, enterococci are often used as an indicator of fecal contamination. We collected samples from four different locations in a commercial beef processing facility in Alberta, Canada, over an 18-month period. Enterococcus spp. were collected from a randomly selected 10- by 10-cm area on carcasses after hide removal (H) and final washing (W), as well as from conveyer belts (C) using a 2- by 2-cm sterile gauze swab (*n* = 150 each). Ground product (*n* = 150) and retail ground beef (*n* = 60) samples (25 g each) were also collected during the same time period. The swabs and ground samples were homogenized and preenriched in a Stomacher 400 circulator (Seward, Norfolk, UK) at 260 rpm for 2 min in 10 ml or 225 ml of buffered peptone water, respectively, and incubated overnight at 37°C. A 1-ml aliquot of this mixture was then added to 9 ml of Enterococcosel broth (BD, Mississauga, Ontario, Canada) and incubated overnight at 37°C to enrich for *Enterococcus* spp. Enterococcosel broth tubes displaying evidence of esculin hydrolysis (black) were streaked onto Enterococcosel agar and incubated at 37°C. After 48 h, the plates were examined for colonies with black zones, which is indicative of esculin hydrolysis. The *groES*-*EL* spacer region of presumptive enterococcal colonies was amplified using the EntES-211-233-F and Ent-EL-74-95-R primers (1), and the product was sequenced using an ABI Prism 3130xl genetic analyzer (Thermo Fisher Scientific, Inc., Mississauga, Ontario, Canada) for confirmation and species identification. From the confirmed *Enterococcus* spp., 36 E. faecalis and 7 E. faecium isolates were selected for whole-genome sequencing.

Briefly, genomic DNA was extracted using the DNeasy blood and tissue kit (Qiagen, Mississauga, Ontario, Canada) with the modification that cells were incubated with agitation (150 rpm) for 45 min at 37°C in 280 μl of lysis buffer (20 mM Tris-HCl [pH 8.0], 2 mM sodium EDTA, 1.2% Triton X-100, and 20 mg/ml lysozyme; Sigma-Aldrich Canada, Toronto, Ontario, Canada). The Nextera XT DNA library preparation kit (Illumina, Inc., San Diego, CA, USA) was used to prepare sequencing libraries that were sequenced on a MiSeq instrument (Illumina, Inc.) with the MiSeq reagent kit v3 (Illumina, Inc.; 600 cycles) as per the manufacturer’s instructions.

FastQC v0.11.5 (2) was used to assess read quality before (678,235 ± 23,253 [standard error of the mean {SEM}] reads per isolate) and after (596,916 ± 21,456 reads per isolate) quality filtering. Sequencing adapters, reads with a quality score of less than 15 over a 4-bp sliding window, and reads that were less than 50 bp in length were removed with Trimmomatic v0.38 (3). The paired-end reads were assembled with SPAdes v3.11.1 (4), with the default parameters in the “careful” mode, and the quality of the assemblies was determined using QUAST v5.0.1 (5). Contigs less than 500 bp in length were removed prior to confirming the taxonomy of each assembly with Kraken 2 v2.0.7-beta and the minikraken2 database v2 (6). The assemblies were then annotated with Prokka v1.13.3 (7) using the default parameters. Multilocus sequence typing (MLST) was done using the Enterococcus faecalis MLST website (https://pubmlst.org/efaecalis/) (8) and the Enterococcus faecium MLST website (https://pubmlst.org/efaecium/) (9). The assembly statistics, GenBank and SRA accession numbers, and MLST results for each isolate are presented in Table 1.

**TABLE 1:** Assembly statistics for Enterococcus faecalis and Enterococcus faecium isolates from a beef processing facility and retail ground beef^*a*^

Isolate name	Species	GenBank accession no.	SRA accession no.	No. of contigs	No. of reads	Genome size (bp)	*N*_50_ value (bp)	Coverage (×)	No. of coding sequences	G+C content (%)	MLST
C112	E. faecalis	GCA_006541215	SRR9321129	26	722,884	2,988,147	605,310	73	2,886	37.43	Unknown
C138	E. faecalis	GCA_006541395	SRR9321128	30	899,074	2,695,188	213,629	100	2,557	37.59	228
C144	E. faecalis	GCA_006541075	SRR9321127	34	689,845	2,697,493	222,684	77	2,519	37.58	228
C146	E. faecalis	GCA_006541305	SRR9321126	28	502,816	2,697,692	351,123	56	2,567	37.59	228
G109	E. faecalis	GCA_006541225	SRR9321133	65	463,324	2,853,332	140,707	49	2,739	37.57	Unknown
G127E	E. faecalis	GCA_006541295	SRR9321132	105	633,257	3,053,018	80,851	62	2,997	37.48	Unknown
G138E	E. faecalis	GCA_006541335	SRR9321131	119	564,994	3,016,964	86,924	56	2,917	37.45	21
G149	E. faecalis	GCA_006541345	SRR9321130	125	446,360	2,994,467	59,749	45	2,854	37.42	40
G42	E. faecalis	GCA_006541405	SRR9321125	97	630,933	2,872,254	73,537	66	2,712	37.56	21
G69E	E. faecalis	GCA_006541355	SRR9321124	17	529,454	2,959,381	645,373	54	2,784	37.51	202
G81	E. faecalis	GCA_006541905	SRR9321137	59	859,935	2,835,934	144,986	91	2,697	37.55	Unknown
G85	E. faecalis	GCA_006541465	SRR9321136	64	640,416	2,876,123	124,461	67	2,732	37.52	76
H102	E. faecalis	GCA_006541755	SRR9321139	106	416,566	2,830,742	73,730	44	2,747	37.6	147
H112E	E. faecium	GCA_006541175	SRR9321138	42	512,378	2,655,194	141,400	58	2,519	38.05	212
H134E	E. faecium	GCA_006541535	SRR9321141	66	535,680	2,507,908	101,754	64	2,365	38.07	29
H136	E. faecalis	GCA_006541265	SRR9321140	22	883,588	3,052,803	378,930	87	3,001	37.34	Unknown
H22	E. faecalis	GCA_006541095	SRR9321143	35	657,288	2,786,536	250,863	71	2,564	37.49	16
H4	E. faecalis	GCA_006541805	SRR9321142	59	641,369	2,996,880	146,743	64	2,860	37.42	40
H44	E. faecalis	GCA_006541185	SRR9321135	46	649,040	2,816,920	228,593	69	2,629	37.57	76
H74	E. faecalis	GCA_006541115	SRR9321134	38	769,279	2,733,857	166,527	84	2,575	37.57	Unknown
H96E	E. faecalis	GCA_006541255	SRR9321160	32	664,950	2,847,191	191,075	70	2,644	37.53	708
R2	E. faecium	GCA_006541615	SRR9321161	145	784,155	2,743,995	46,108	86	2,682	38.24	76
R20	E. faecalis	GCA_006541155	SRR9321162	163	623,226	2,985,199	78,020	63	2,859	37.52	260
R26E	E. faecium	GCA_006541645	SRR9321163	237	261,875	2,688,512	23,732	29	2,594	38.38	76
R29	E. faecalis	GCA_006541545	SRR9321156	45	468,536	2,911,452	206,037	48	2,785	37.47	260
R30	E. faecalis	GCA_006541705	SRR9321157	49	505,869	2,647,103	84,312	57	2,488	37.7	228
R37	E. faecalis	GCA_006541775	SRR9321158	70	482,350	2,929,864	135,712	49	2,769	37.39	260
R43E	E. faecalis	GCA_006541085	SRR9321159	73	591,636	2,833,916	87,778	63	2,734	37.49	Unknown
R49	E. faecalis	GCA_006541715	SRR9321154	36	390,483	3,029,112	153,103	39	2,921	37.37	192
R4E	E. faecium	GCA_006541685	SRR9321155	100	667,395	2,738,432	75,527	73	2,683	38.24	Unknown
R50	E. faecalis	GCA_006541785	SRR9321147	113	519,617	2,976,798	77,751	52	2,863	37.46	21
R51	E. faecalis	GCA_006541855	SRR9321146	62	463,047	3,005,276	121,849	46	2,957	37.45	84
R52	E. faecalis	GCA_006541795	SRR9321145	66	449,634	3,046,695	119,574	44	2,931	37.37	260
R53	E. faecalis	GCA_006541655	SRR9321144	79	596,560	3,030,982	119,130	59	2,966	37.42	260
R5E	E. faecalis	GCA_006541525	SRR9321151	76	611,462	2,934,492	97,444	63	2,785	37.39	Unknown
R7	E. faecalis	GCA_006541595	SRR9321150	51	507,624	2,911,924	184,949	52	2,821	37.47	260
W100	E. faecalis	GCA_006541515	SRR9321149	55	452,646	2,961,448	130,717	46	2,832	37.42	260
W133	E. faecalis	GCA_006541445	SRR9321148	364	678,090	2,944,917	14,728	69	2,799	37.19	Unknown
W141	E. faecium	GCA_006541625	SRR9321153	82	626,699	2,761,265	123,573	68	2,695	38.22	76
W148E	E. faecium	GCA_006541485	SRR9321152	96	501,112	2,758,663	89,896	54	2,687	38.23	76
W19	E. faecalis	GCA_006541875	SRR9321122	76	865,657	2,956,998	112,037	88	2,846	37.5	Unknown
W84	E. faecalis	GCA_006541885	SRR9321123	99	564,979	3,018,223	89,324	56	2,896	37.45	Unknown
W97	E. faecalis	GCA_006541435	SRR9321121	61	741,306	3,050,168	155,000	73	2,961	37.37	40

a The number of coding sequences is based on Prokka annotations. Annotations in GenBank are based on the Prokaryotic Genome Annotation Pipeline (PGAP).

The genome assemblies were also screened for the presence of antimicrobial resistance (AMR) genes using BLASTn 2.6.0+ and the Comprehensive Antibiotic Resistance Database (CARD; v3.0.2) (10), with a minimum identity of 90%. The most prevalent AMR genes in the E. faecalis genomes were *tet*(M) (25%) and *erm*(B) (8%); in the E. faecium assemblies, *msrC* (100%), *aac(6′)-II* (100%), and *tet*(M) (29%) were most frequently detected.

### Data availability.

All sequences and draft genome assemblies have been deposited in the Sequence Read Archive and GenBank, respectively, under the accession numbers listed in Table 1.
